# Supplementary material for: Emergence of cortical network motifs for short-term memory during learning
Source: Nat Commun. 2023 Oct 28;14:6869. doi: 10.1038/s41467-023-42609-4 (PMC10613236; doi:10.1038/s41467-023-42609-4)
Supplement: Supplementary file 1 — Supplementary Information [file 41467_2023_42609_MOESM1_ESM.pdf]

# Supplementary Information

## Emergence of cortical network motifs for short-term memory during learning

Xin Wei Chia[1], Jian Kwang Tan[1,2], Lee Fang Ang[1,2], Tsukasa Kamigaki[1], and Hiroshi Makino[1]*
*Correspondence: hmakino@ntu.edu.sg

[1]Lee Kong Chian School of Medicine, Nanyang Technological University
Singapore, 308232, Singapore
[2]These authors contributed equally.

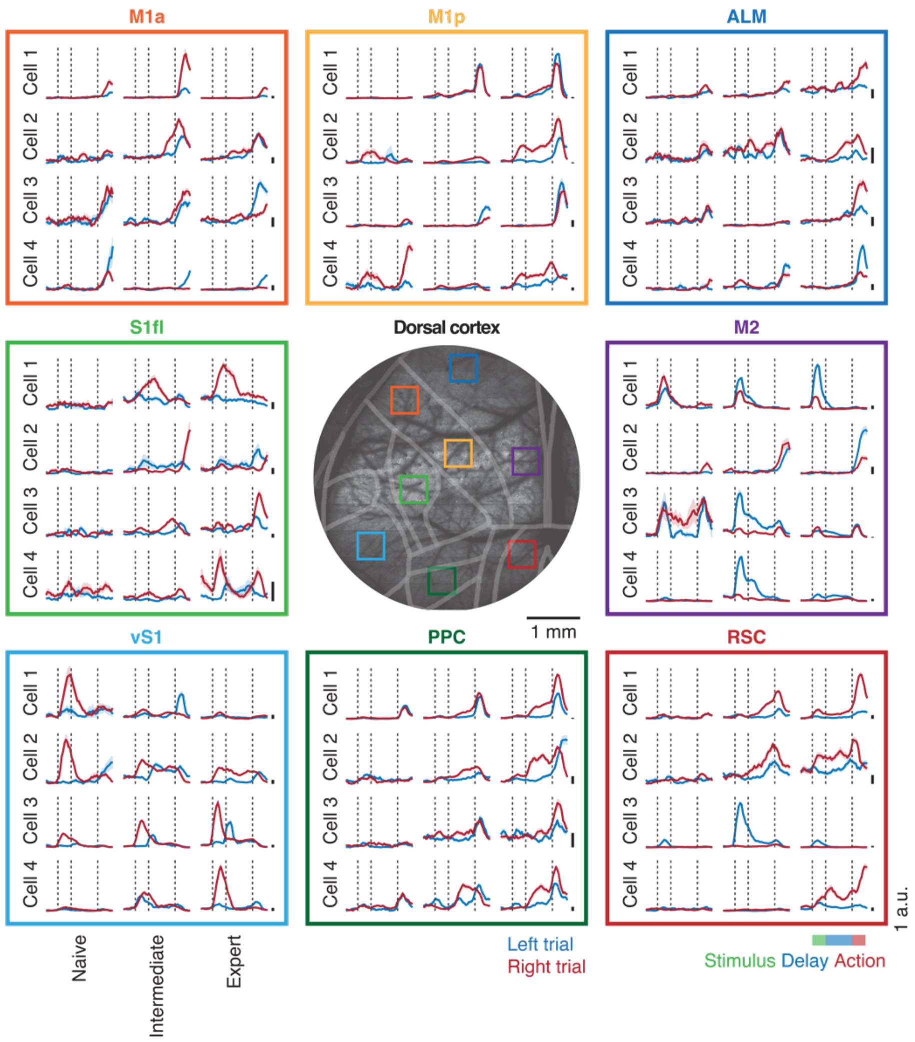


**Figure S1. Changes in task-related activity of example neurons across learning in each cortical region.**
Colors denote different cortical regions. Shaded area indicates mean ± SEM.

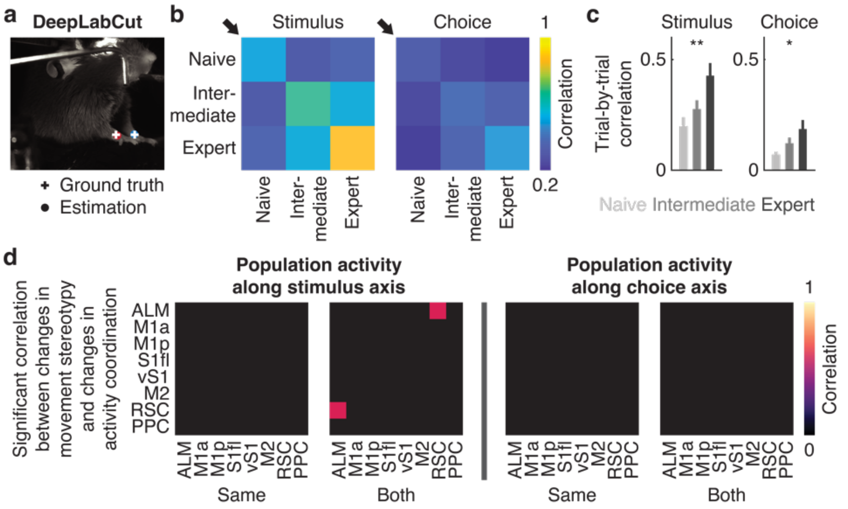


**Figure S2. Relationship between changes in movement stereotypy and changes in intra- and inter-regional coordination of sensorimotor representation across learning.**

**(a)** Example video frame analyzed with DeepLabCut illustrating the ground truth and estimated forelimb positions.

**(b)** Trial-by-trial correlations of right forelimb movements across the three learning stages. Arrows indicate diagonal entries of the matrices.

**(c)** Within-session trial-by-trial correlations of right forelimb movements across learning, corresponding to the arrows on left panels (**P = 0.003, *P = 0.03, stimulus: 13 sessions from 7 mice; choice: 13 sessions from 7 mice, one-way ANOVA). Error bars indicate mean ± SEM.

**(d)** Correlations between changes in movement stereotypy and changes in trial-by-trial correlations of population activity along the stimulus and choice axis. Only significant correlations with P < 0.05 (Pearson correlation) are shown. Note that PPC-ALM shows no statistically significant relationships. Same: same trial types; Both: both trial types.

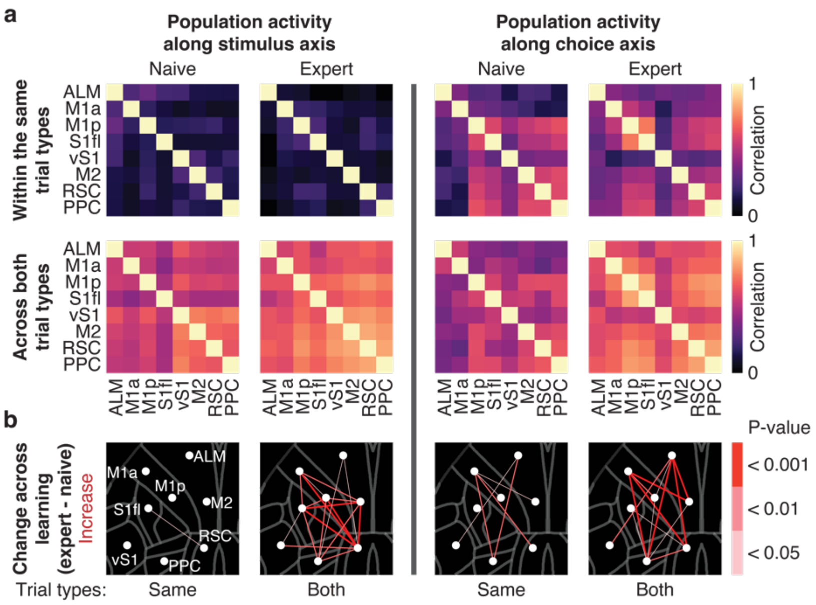


**Figure S3. Intra- and inter-regional coordination of sensorimotor representation across learning after balancing the number of trial types.**

**(a)** Top. Trial-by-trial correlations of population activity within the same trial types along the stimulus (left) and choice (right) axis across learning after balancing the number of left and right trial types. Bottom. Same as above but for both trial types.

**(b)** P-values for increases in the correlation coefficients across learning in **(a)** with respect to the location of each cortical region (one-tailed bootstrap). Same: same trial types; Both: both trial types.

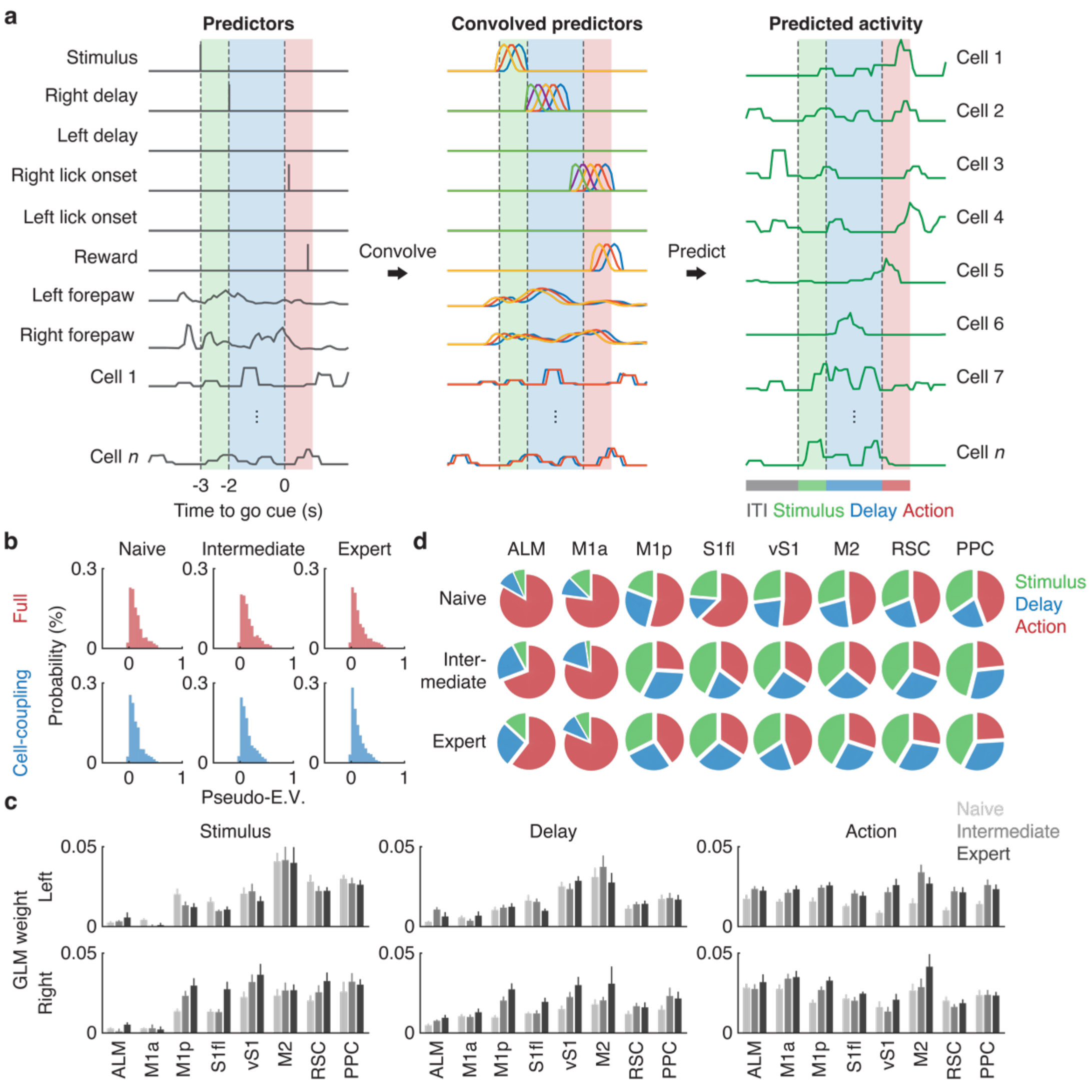


**Figure S4. Generalized linear model (GLM).**

**(a)** Schematic of GLM. Task variables were convolved with basis functions in time and the model was trained to fit the deconvolved calcium signal for each neuron by regression.

**(b)** Probability distributions of pseudo-E.V. in full and cell-coupling models across learning.

**(c)** Region-specific GLM weights for individual task variables (ALM: 7; M1a: 10; M1p: 11; S1fl: 8; vS1: 12; M2: 11; RSC: 13; PPC: 13 sessions from 7 mice for naive, intermediate and expert). Error bars indicate mean ± SEM.

**(d)** Relative fractions of stimulus-, delay- or action-encoding neurons in each cortical region across learning.

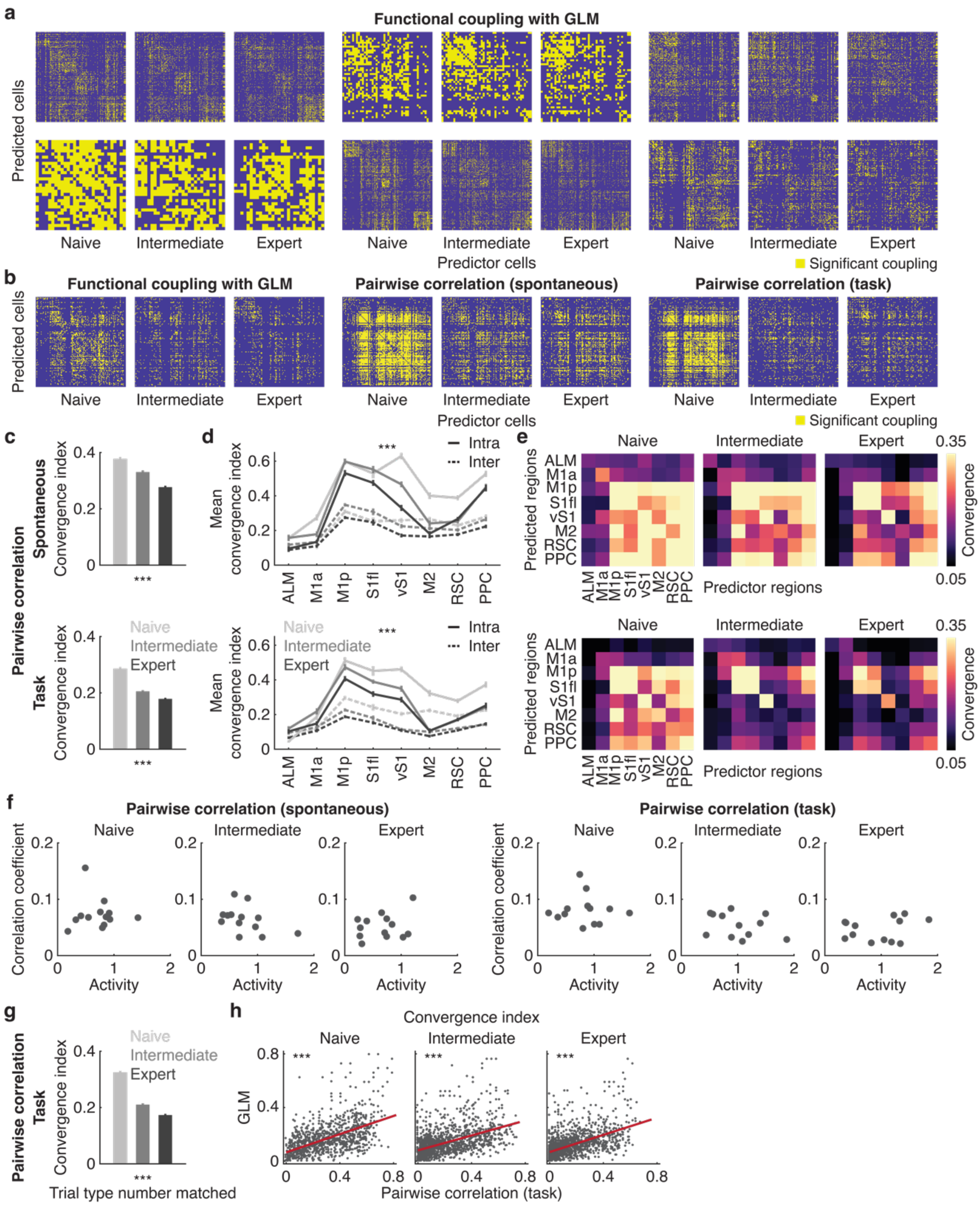


**Figure S5. Additional analysis for sparsening of functional connectivity across learning.**

**(a)** Sparsening of functional coupling across learning in example sessions. Each yellow box indicates a significant functional coupling from a predictor to a predicted cell. Note that the different pixel size reflects different number of neurons.

**(b)** Sparsening of functional connectivity measured by three independent methods. Spontaneous and task indicate correlations of ITI and pre-action activity between pairs of neurons, respectively.

**(c)** Decrease in convergence index in the dorsal cortex across learning measured by pairwise correlations of ITI (top) and pre-action (bottom) activity (***$P < 0.001$, $n = 1475$ neurons from 7 mice, one-way ANOVA). Error bars indicate mean ± SEM.

**(d)** Intra- and inter-regional convergence index across learning derived from ITI (top) and pre-action (bottom) activity (***$P < 0.001$ for main effects of intra- versus inter-region, across learning and across regions, ALM: 82; M1a: 123; M1p: 211; S1fl: 177; vS1: 205; M2: 94; RSC: 287; PPC: 242 neurons from 7 mice, three-way ANOVA). Error bars indicate mean ± SEM.

**(e)** Mean convergence index across the dorsal cortex at the three learning stages derived from ITI (top) and pre-action (bottom) activity.

**(f)** Pairwise correlations of ITI (left) and pre-action (right) activity were not related to activity levels at each learning stage.

**(g)** Decrease in convergence index in the dorsal cortex across learning, measured by pairwise correlations of pre-action ("task") activity after balancing the number of left and right trial types (***$P < 0.001$, n = 1475 neurons from 7 mice, one-way ANOVA). Error bars indicate mean ± SEM.

**(h)** Positive relationship between convergence index obtained in **(g)** and convergence index obtained from GLM (naive: $R^2 = 0.27$, ***$P < 0.001$; intermediate: $R^2 = 0.23$, ***$P < 0.001$; expert: $R^2 = 0.25$, ***$P < 0.001$, 1475 neurons from 7 mice, Pearson correlation).

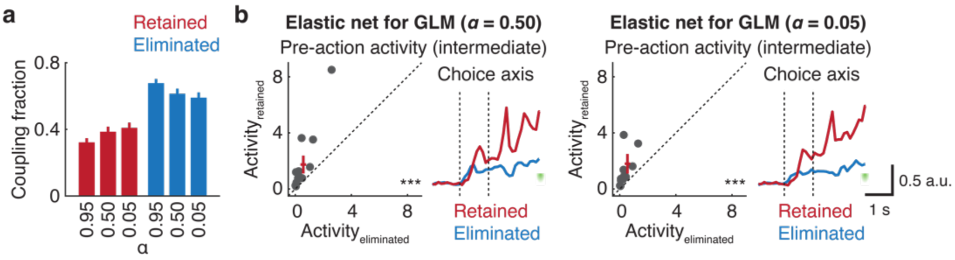


**Figure S6. Selection of functional coupling containing choice information across learning with different $\alpha$ values of elastic net regularization.**

**(a)** Fractions of retained and eliminated functional coupling from the intermediate to expert stage across different $\alpha$ values (n = 13 sessions from 7 mice). Error bars indicate mean ± SEM.

**(b)** Left. Choice selectivity computed by averaging pre-action population activity projected to the choice axis with $\alpha$ = 0.50 (1 s in duration, starting 1 s prior to the go cue, ***P < 0.001, n = 13 sessions from 7 mice, one-tailed bootstrap). Red cross indicates mean ± SEM. Inset: session-averaged reconstructed population activity prior to the go cue with retained (red) or eliminated (blue) coupling projected to the choice axis. Right. Same as left with $\alpha$ = 0.05.

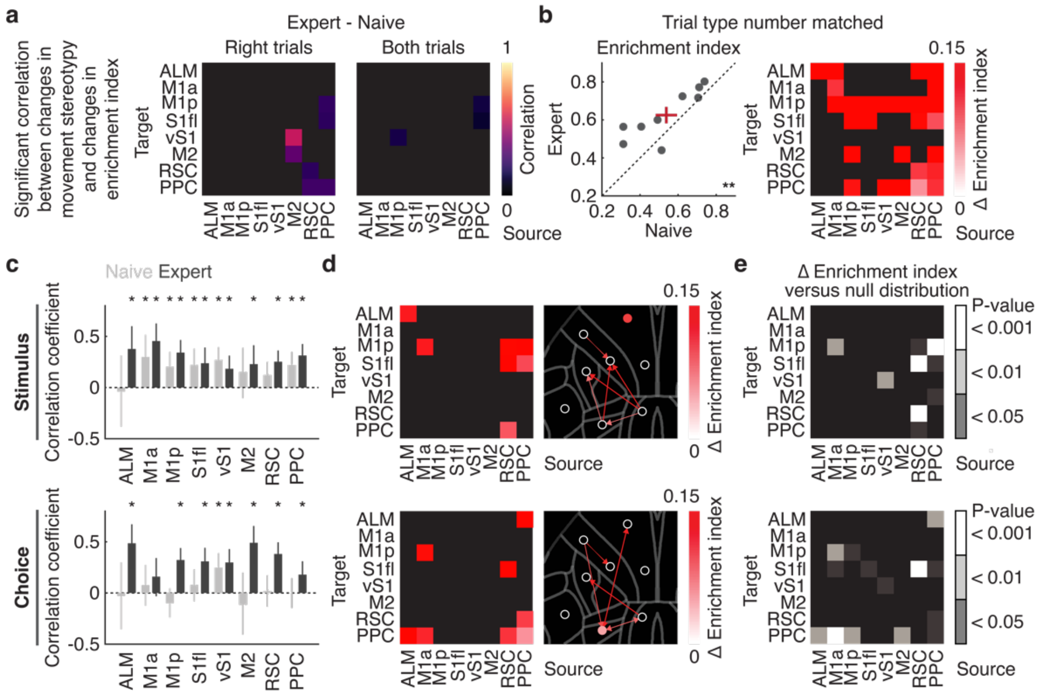

**Figure S7. Additional analysis for enrichment index.**

**(a)** Correlations between changes in movement stereotypy and changes in enrichment index for right and both trial types. Only significant positive correlations with P < 0.05 (Pearson correlation) are shown. Note that PPC-ALM shows no statistically significant relationships.

**(b)** Left. Changes in enrichment index of choice-encoding cells across learning after balancing the number of left and right trial types (**P = 0.003, n = 9 sessions from 6 mice, one-tailed bootstrap). Red cross indicates mean ± SEM. Right. Learning-related increases in enrichment index for right-preferring choice-encoding cells without accounting for random changes in functional coupling. Only pairs of regions with P < 0.05 (one-tailed bootstrap) are displayed.

**(c)** Region-specific correlation coefficients between stimulus (top)- or choice (bottom)-selectivity and enrichment index across learning. Error bars indicate 95% confidence intervals from the mean (*P < 0.05, stimulus: ALM: 47; M1a: 67; M1p: 180; S1fl: 134; vS1: 206; M2: 95; RSC: 266; PPC: 230 neurons from 7 mice; choice: ALM: 51; M1a: 105; M1p: 206; S1fl: 163; vS1: 169; M2: 67; RSC: 193; PPC: 201 neurons from 7 mice, Pearson correlation with FDR).

**(d)** Left. Learning-related increases in enrichment index for right-preferring stimulus (top)- and choice (bottom)-encoding cells before accounting for random changes in functional coupling, which is described in **(e)**. Only pairs of regions with P < 0.05 (one-tailed bootstrap) are displayed. Right. Changes in enrichment index with respect to the location of each region. Color of solid circles indicates intra-regional enrichment index. Thickness of lines is proportional to inter-regional enrichment index.

**(e)** P-values of changes in correlation coefficients for stimulus (top)- and choice (bottom)-encoding neurons across learning when compared to a null distribution (one-tailed bootstrap). Statistically significant changes indicate those that are not explained by random changes in the fractions of stimulus- or choice-encoding neurons.

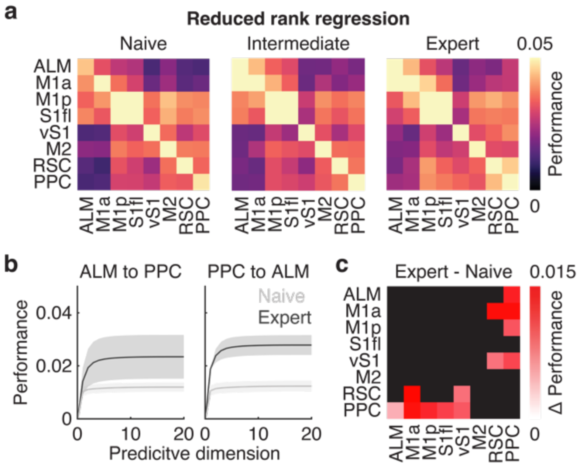


**Figure S8. Intra- and inter-regional communications analyzed by reduced rank regression.**

**(a)** Reduced rank regression to predict the population activity of a target region based on population activity of a source region. Five dimensions were used to assess the prediction performance at each learning stage.

**(b)** Prediction performance as a function of the number of predictive dimensions for ALM-PPC and PPC-ALM interactions at the naive and expert stage (n = 20 shuffles). Shaded areas indicate mean ± SEM.

**(c)** Changes in the prediction performance across learning. Only pairs of regions with $P < 0.05$ (one-tailed bootstrap) are displayed.

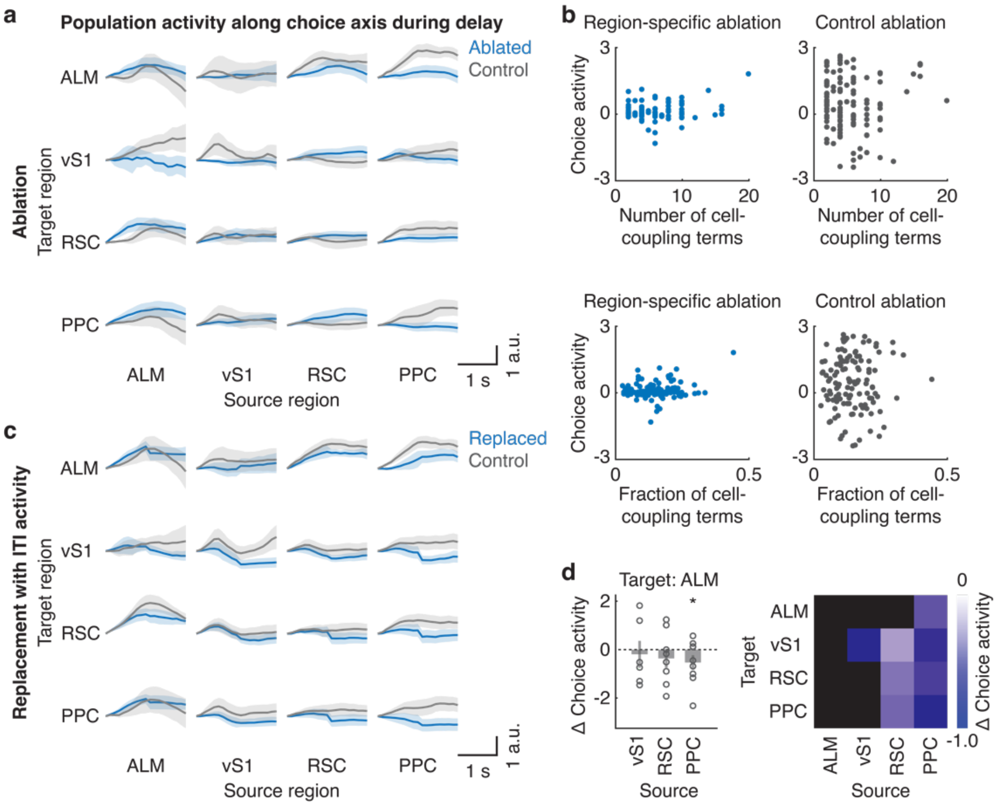


**Figure S9. Population activity along the choice axis following targeted and control ablation.**

**(a)** Session-averaged reconstructed population activity with functional coupling following targeted (blue) and control ablation (gray) of cell-coupling terms projected to the choice axis during the delay epoch across different region pairs (n = 14 sessions from 7 mice). Shaded area indicates mean ± SEM.

**(b)** Top. Correlations between the median numbers of cell-coupling terms used to reconstruct the choice activity and mean changes in choice activity across region pairs analyzed in **(a)** (region-specific ablation: n.s., P = 0.09; control ablation: n.s., P = 0.52, two-tailed bootstrap with Pearson correlation). Bottom. Same as above but with median fractions of cell-coupling terms (region-specific ablation: n.s., P = 0.88; control ablation: n.s., P = 0.63, two-tailed bootstrap with Pearson correlation)**.**

**(c)** Same as **(a)** but with replacement of the pre-action activity with scrambled ITI activity. Shaded area indicates mean ± SEM.

**(d)** Left. Differences between choice-related population activity following targeted and control replacement averaged within the pre-action epoch (*P < 0.05, vS1: 6; RSC: 8; PPC: 9 sessions from 6 mice, one-tailed bootstrap with FDR). Error bars indicate mean ± SEM. Right, Same as left but for additional pairs of regions that showed statistically significant changes (one-tailed bootstrap).

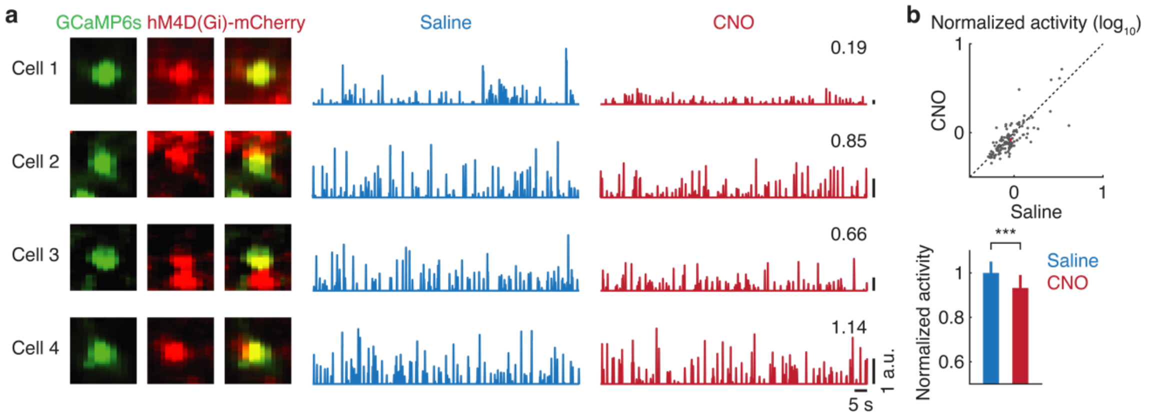


**Figure S10. PPC suppression with the DREADDs experiment.**

**(a)** Spontaneous activity of example neurons expressing GCaMP6s and hM4D(Gi)-mCherry with saline and CNO injection. The numbers indicate the activity with CNO injection normalized to the activity with saline injection.

**(b)** Suppression of PPC spontaneous activity. The activity with CNO injection was normalized to the activity with saline injection (***P < 0.001, n = 119 neurons from 2 mice, two-tailed Wilcoxon signed-rank test). Red cross indicates mean ± SEM. Error bars indicate mean ± SEM.

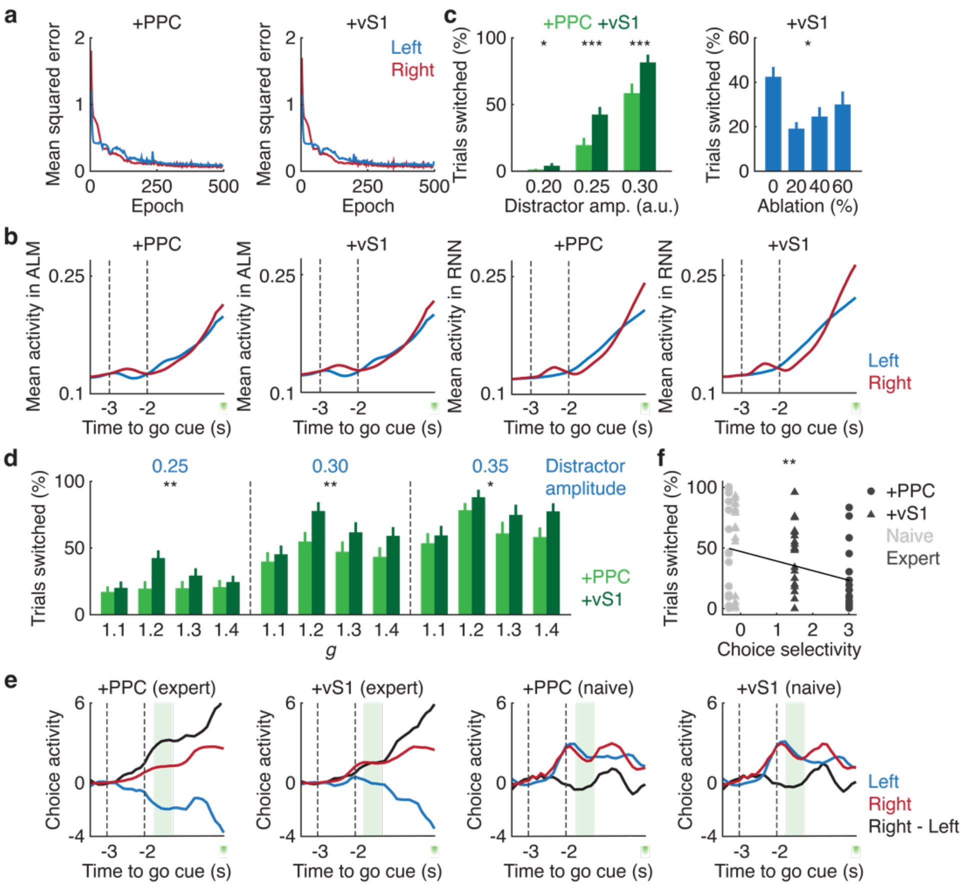

**Figure S11. RNN training and parameter dependency.**

**(a)** Examples of mean squared errors between normalized activity of ALM neurons and RNN units during training with PPC-ALM (+PPC) and vS1-ALM (+vS1) external activity.

**(b)** Left. Examples of average normalized population activity of ALM neurons reconstructed with PPC-ALM (+PPC) and vS1-ALM (+vS1) external activity. Right. Examples of average population activity of RNN units trained to reproduce the ALM activity with PPC-ALM (+PPC) and vS1-ALM (+vS1) external activity.

**(c)** Left. Fractions of decision-switching trials as a function of distractor amplitude (n = 21 RNNs, ***P < 0.001 and *P < 0.05, one-tailed bootstrap with FDR). Stronger distractor caused higher frequency of decision switching for both classes of RNNs. Right. Fractions of decision-switching trials as a function of percentage of ablated connections in RNNs trained with vS1-ALM external activity (n = 21 RNNs, *P = 0.01, one-way ANOVA). Error bars indicate mean ± SEM.

**(d)** Same as **(c)** but with different values of $g$ and distractor amplitude (**P = 0.008, **P = 0.005 and *P = 0.02 for a main effect between PPC and vS1 with distractor amplitude of 0.25, 0.30 and 0.35, respectively, n = 33, 21, 25 and 30 RNNs for $g$ = 1.1, 1.2, 1.3 and 1.4, respectively, three-way ANOVA). Error bars indicate mean ± SEM.

**(e)** External coupling activity of PPC-ALM or vS1-ALM neurons projected to the choice axis across learning (PPC: 171 neurons from 4 mice; vS1: 131 neurons from 4 mice). Shaded green rectangle denotes the distraction epoch.

**(f)** Correlation between fractions of decision-switching trials and average choice selectivity from **(e)** during the distractor epoch (**P = 0.005, PPC: 20 and 21; vS1: 20 and 21 RNNs for naive and expert, respectively, Pearson correlation).
